# Six Persistent Research Misconceptions

**DOI:** 10.1007/s11606-013-2755-z

**Published:** 2014-01-23

**Authors:** Kenneth J. Rothman

**Affiliations:** 1 Research Triangle Institute, Research Triangle Park, NC USA; 2 Boston University School of Public Health, Boston, MA USA

**Keywords:** study design, data interpretation, epidemiologic methods, representativeness, evaluation of interaction, multiple comparisons, percentile boundaries, statistical significance testing

## Abstract

Scientific knowledge changes rapidly, but the concepts and methods of the conduct of research change more slowly. To stimulate discussion of outmoded thinking regarding the conduct of research, I list six misconceptions about research that persist long after their flaws have become apparent. The misconceptions are: 1) There is a hierarchy of study designs; randomized trials provide the greatest validity, followed by cohort studies, with case–control studies being least reliable. 2) An essential element for valid generalization is that the study subjects constitute a representative sample of a target population. 3) If a term that denotes the product of two factors in a regression model is not statistically significant, then there is no biologic interaction between those factors. 4) When categorizing a continuous variable, a reasonable scheme for choosing category cut-points is to use percentile-defined boundaries, such as quartiles or quintiles of the distribution. 5) One should always report P values or confidence intervals that have been adjusted for multiple comparisons. 6) Significance testing is useful and important for the interpretation of data. These misconceptions have been perpetuated in journals, classrooms and textbooks. They persist because they represent intellectual shortcuts that avoid more thoughtful approaches to research problems. I hope that calling attention to these misconceptions will spark the debates needed to shelve these outmoded ideas for good.

A surprising number of misconceptions persist in the conduct of research involving human subjects. Some persist despite teachings to the contrary, and some because of teachings that should be to the contrary. To spark discussion of these issues, I list here six persistent research misconceptions, and offer a capsule summary of the problems with each of them.

Misconception 1. *There is a hierarchy of study designs; randomized trials provide the greatest validity, followed by cohort studies, with case–control studies being least reliable*.

Randomized trials, though often considered the “gold standard” of study types, are not perfect, even in concept. Furthermore, the premise that the comparative validity of study results can be inferred from the type of study is wrong.

Although some believe that evidence from a randomized trial is as compelling as a logical proof, no empirical finding can provide absolute certainty. If randomized trials were perfect, how could they give divergent results? In fact, they are subject to various errors.^1^ Obviously there is random error, as one would expect from a study based on random assignment. But there is also systematic error, or bias. For example, randomized trials are usually analyzed using the “intent to treat” principle, which compares the groups that are initially assigned by randomization, regardless of any subsequent non-adherence. Non-adherence results in underestimation of any treatment effect. This bias is usually considered acceptable because it is outweighed by the advantages achieved by random assignment. Underestimation of effects, however, is not acceptable in a safety trial aimed at uncovering adverse effects of the treatment. Another important source of bias in a randomized trial comes from errors in assessing the outcome, such as undercounting of outcome events. Also, even if randomization provides a balance of risk factors between groups at the start of the trial, with extended follow-up, the study groups may become progressively imbalanced through differential attrition or changes in risk factor distributions. With long-term trials, the benefits of random assignment may therefore fade with time.

In short, trials are far from perfect. Furthermore, both cohort and case–control studies will yield valid results when properly designed and carried out. Therefore, mindlessly ascribing greater validity to a study based on a hierarchy of designs^2^,^3^ is fallacious. For example, the relation between cigarette smoking and lung cancer is well established, based on findings from cohort and case–control studies. The connection was never shown clearly in a randomized trial. It is not easy to assign people randomly to smoke or not smoke; however, when smoking cessation was studied as part of a multi-pronged intervention in the randomized Multiple Risk Factor Intervention Trial,^4^ those who were urged to cease smoking actually developed more lung cancer than those who did not receive the cessation encouragement. The results of the trial did not overthrow the findings of the many cohort and case–control studies conducted without randomization. Rather, the discrepancy was ascribed to problems with the trial.

In another high-profile example, results from large cohort studies^5^,^6^ indicated that risk of coronary heart disease was reduced among postmenopausal hormone users, but later results from two randomized trials indicated either no association or an increased risk.^7^,^8^ The reaction in the scientific community and the popular press^9^ was to discredit the results from the cohort studies, presuming that they had been refuted by the randomized trials. Many continue to believe that interpretation, but in an elegant reanalysis, Hernan et al.^10^ showed that the study populations in the cohort studies and the randomized trials were different, and that the effects of postmenopausal hormone use varied greatly according to age and time since menopause. When studies were restricted to new users of hormones, Hernan et al. showed that differences in the distribution of age and time since menopause could explain all of the apparent discrepancies. Although it is common to ascribe such discrepancies to inherent weaknesses of the nonexperimental studies, it is simplistic to assign validity based on a presumed hierarchy of study types.^11^

Similarly, discrepancies between cohort studies and case–control studies should not be explained away superficially by a presumed validity advantage for cohort studies over case–control studies. Properly designed case–control studies will produce the same results as properly designed cohort studies. When conflicts arise, they could stem from problems in either or both types of study. Although case–control studies have long been disparaged as being backwards versions of cohort studies, starting from disease and tracing back to possible causes, epidemiologists today understand case–control studies to be conceptually identical to cohort studies, apart from an efficiency gain that comes from sampling the denominators rather than conducting a complete census. Indeed, the efficiency gain may allow more resources for exposure assessment or case validation in case–control studies, resulting in less bias than in corresponding cohort studies of the same relation.

Those who view case–control studies as backwards versions of cohort studies sometimes make the false analogy that the controls should closely resemble the cases, except that they lack the case-defining disease. In fact, the control group in a case–control study is intended to be a sample of the population denominator that gives rise to the cases, a substitute for the full denominators obtained in a cohort study. Thus, the control group should resemble the entire study population, rather than the cases.^12^,^13^ When properly designed, case–control studies can achieve the same excellent validity as properly designed cohort studies, whereas a poorly designed trial can be unreliable. The type of study should not be taken as a guide to a study’s validity.

Misconception 2. *An essential element of making valid generalizations from a study is that the study subjects constitute a representative sample of a target population.*

This misconception is tied to the view that scientific generalization involves the mechanical extrapolation of results from a sample to its source population. But that describes statistical generalization; scientific generalization is different: it is the process of constructing a correct statement about the way nature works.

Scientific generalization is the ultimate goal of scientific inquiry, but a prerequisite is designing a study that has internal validity, which is enhanced by keeping all disturbing variables constant. When have we heard of animal researchers who seek a statistically representative sample of animals? Instead, their operating principle is nearly the opposite of seeking representativeness. Thus, biologists studying mice prefer to study mice that are homogeneous with respect to genes and environment, and that differ only in respect to the experimentally manipulated variable. Unlike the statistical generalization of opinion polls or survey sampling, which merely calls for extrapolation from sample to source population, scientific generalization proceeds by informed guesses, but only from the secure platform of a valid study. Consequently, studies are stronger if they limit variability of confounding factors, as opposed to seeking representativeness. Doll and Hill^14^ studied the mortality of male British physicians in relation to their smoking habits. Their findings were considered broadly generalizable despite the fact that their study population was unrepresentative of the general population of tobacco users with regard to sex, race, ethnicity, social class, nationality and many other variables.

When there is a legitimate question about whether an overall association varies by subgroup of some third variable, such as age or ethnic group, it may be necessary to include people drawn from a broad range of values of that third variable, but even then it is counterproductive for the study population to be representative of the source population for that variable. The goal in that case would be to include study subjects distributed evenly across the range, or in a distribution that enhances overall study efficiency. A sample that is representative of the source population will be suboptimal.^15^,^16^

Misconception 3. *If a term that denotes the product of two factors in a regression model is not statistically significant, then there is no biologic interaction between those factors.*

“Biologic” is meant here broadly, to encompass biochemical, psychological, behavioral and physical interactions. The problem is that interaction is usually evaluated through regression models, in which the product term addresses statistical interaction rather than biologic interaction.

Biologic interaction refers to two or more causes acting in the same mechanism, with effects that are mutually dependent. It describes a state of nature. If basic effects are measured as changes in disease risk, synergistic (i.e. positive) biologic interaction is present when the joint effect of two causal factors is more than the sum of their effects acting separately.^17^ In contrast, statistical interaction does not describe nature; it describes a mathematical model. It is typically assessed with a product term for two variables in a regression model. Its magnitude depends on the choice of measures and scale of measurement. Statistical interaction implies only that the basic functional form of a specific mathematical model is not an apt description of the relation among variables. Two factors that show biologic interaction may or may not exhibit statistical interaction, depending on the model used.

Product terms in regression models have units that can defy interpretation. If one variable is fat consumption, measured in grams per day, and another variable is pack-years of cigarettes smoked, what is the interpretation of a variable that has units of grams/day multiplied by pack-years? The challenge of interpreting such product term coefficients has fostered a focus on the p value accompanying the coefficient, rather than the magnitude of the coefficient itself. Focusing on the pvalue, or on whether the coefficient of a product term is statistically significant, only worsens the problem of mistaking statistical interaction for biologic interaction (see misconception 6). A more meaningful assessment of interaction would be to focus on the proportion of cases of a disease that one could attribute to biologic interaction.^17^,^18^

Consider a simple example from the TREAT trial (Trial to Reduce Cardiovascular Events with Aranesp Therapy),^19^ which evaluated the risk of stroke among 4,038 patients with diabetes mellitus, chronic kidney disease, and anemia randomized to receive darbepoetin alfa or placebo. Among patients without a history of stroke, the risk of stroke during the study period was 2 % among patients receiving placebo and 4 % among patients receiving darbepoeitin alfa. Among patients with a history of stroke, the corresponding risks were 4 % and 12 %. The authors noted that the risk increase was greater for darbepoeitin alfa among those with a history of stroke, but they dismissed this interaction because the product term in a logistic regression model was not statistically significant. The increased risk attributable to darbepoeitin alfa was 2 % in the patients without a history of stroke and 8 % among patients with a history of stroke, indicating strong biologic interaction between darbepoeitin alfa and history of stroke. If the risks were merely additive, the risk would be 6 % among those with both risk factors, instead of the actual 12 %. Thus, half of the risk among those with both risk factors appears attributable to biologic interaction, despite the authors’ claim that there was no interaction.

Misconception 4. *When categorizing a continuous variable, a reasonable scheme for choosing category cut-points is to use percentile-defined boundaries, such as quartiles or quintiles of the distribution.*

There are two reasons why using percentiles is a poor method for choosing category boundaries. First, these boundaries may not correspond to the parts of the distribution where biologically meaningful changes occur. Suppose you were conducting a study of vitamin C intake and scurvy risk in the U.S. If you decided to categorize vitamin C intake by quintiles, you would find that the entire relation between vitamin C consumption and scurvy was confined to the lowest quintile, and within that category, to only a small proportion of people who were outliers in their low vitamin C intake. 10 mg/day of vitamin C can prevent scurvy, but those consuming less than that represent a fraction of 1 % of the population in the U. S.^20^ Using percentile-based categories would make it impossible to find the effect of inadequate vitamin C intake on scurvy risk, because all intake above 10 mg/d is essentially equivalent. If we routinely use percentile cut-points, we may not know if we are facing the same problem as we would face in the study of vitamin C and scurvy. A more effective alternative would be to begin with many narrow categories, merging neighboring categories until meaningful breaks in risk become evident.

The second problem with percentile-based categories is the difficulty in comparing results across studies, because categories across studies using percentile category boundaries are unlikely to correspond. This problem can be averted by expressing boundary points in terms of the natural units of the variable (such as mg/d for vitamin C intake). It is also useful to report within-category means or medians.

Misconception 5. *One should always report P values or confidence intervals that have been adjusted for multiple comparisons.*

Traditional adjustments for multiple comparisons involve inflating the P value or the width of a confidence interval according to the number of comparisons conducted. If one is analyzing biological data that are replete with actual associations, the premise for traditional adjustments is shaky and the adjustments are difficult to defend. The concern for multiple comparisons stems from fear of finding falsely significant findings (type I errors in the lingo of statistics). In misconception 6, we discuss the problems with using statistical significance testing for data analysis in the first place. But before considering those problems, let us consider the rationale for adjusting reported results for multiple comparisons.

Despite the fact that a single significance test is intended to have a 5 % probability (at the conventionally used level) of being significant when the null hypothesis is true, and therefore multiple tests when properly carried out should each have this property, there is a concern that when making multiple tests, the probability of a spurious result is increased. Of course, as the number of tests increases, the probability that one or more of them would be falsely positive increases, but that is only because many tests are being conducted. Adjustments for multiple comparisons will reduce these type I errors, but they do so at the expense of increasing type II errors, which are nonsignificant test results in the presence of a real association. When observed associations are all the result of chance, type I errors can occur, but type II errors cannot occur. Conversely, when the observed associations all reflect actual relationships, type II errors can occur, but type I errors cannot. Thus, the context of any analysis has fundamental implications regarding the interpretation of the data. In particular, it is absurd to make adjustments that reduce type I errors at the expense of increasing type II errors without some evaluation of the estimated relative cost and frequency of each type of error.

If scientists were put to work studying random numbers instead of biologic data, all the significant results they reported would represent type I errors, and adjustments for multiple comparisons would make sense; some skeptics believe that studies of genome-wide association scans may approximate this situation.^21^ But when scientists are studying biological relations rather than random numbers, the premise that type I errors are the major concern may be wrong.^22^ A more rigorous evaluation of the need for multiplicity adjustments would begin with an assessment of the tenability of the thesis that the data are essentially random numbers. If one is studying experiments on psychic phenomena, skepticism about the results might lend support to multiplicity adjustments. If one is studying physiologic effects of pharmaceutical agents, real associations are to be expected and the adjustments are more difficult to defend. Studying single nucleotide polymorphisms in relation to a given disease might be a middle ground. One approach to this issue that is theoretically more defensible is a Bayesian approach, which assigns prior credibility to various levels of association and adjusts by using Bayes’ theorem to calculate posterior credibility.^23^,^24^

Misconception 6. *Significance testing is useful and important for the interpretation of data*.

Significance testing has led to far more misunderstanding and misinterpretation than clarity in interpreting study results.^25^–^28^ A significance test is a degraded version of the P value, a statistic that blends precision with effect size, thus confusing two essential aspects of data interpretation. Measuring effect size and its precision as separate tasks is a more direct and clearer approach to data interpretation.

For research studies that aim to measure associations, and infer whether they reflect causal connections, focusing on the magnitude of these associations ought to be the primary goal: estimation of effects is decidedly preferable to statistical testing. Ideally, a study estimates the magnitude of the effect size, and analyzes the possible errors that might have distorted it. Systematic errors such as confounding from measured factors can be dealt with through analytic methods; other systematic errors, such as the effects of measurement error or selection bias, can be addressed through sensitivity analyses (also known as bias analysis). Random error is typically expressed through confidence intervals, giving a range of parameter values that are consistent with the data to a specified level.

It is unfortunate that a confidence interval, from which both an estimate of effect size and its measurement precision can be drawn, is typically used merely to judge whether it contains the null value or not, thus converting it to a significance test. Significance tests are a poor classification scheme for study results; strong effects may be incorrectly interpreted as null findings because authors fallaciously interpret lack of statistical significance to imply lack of effect, or weak effects may be incorrectly interpreted as important because they are statistically significant. Rather than be used as surrogate significance tests, confidence intervals ought to be interpreted as quantitative measures indicating magnitude of effect size and degree of precision, with little attention paid to the precise location of the boundaries of the confidence interval. This advice is backed by the Uniform Requirements for Manuscripts Submitted to Biomedical Journals, but nevertheless often overlooked even by reviewers and editors whose journals support the requirements.^29^

Many misconceptions derive from reliance on statistical significance testing. The focus on the statistical significance of interaction terms instead of measuring interaction, as discussed above, is one example. The evaluation of dose–response trends simply by declaring that there is or is not a significant trend, rather than expressing the magnitude and ideally the shape of that trend, is another. Yet another is the advice sometimes offered to calculate the power of a study when reporting results, especially if those results are not statistically significant. Reporting the power of a study as part of the results is called “post-hoc” power calculation.^30^ Power calculations are based on a hypothesis about the level of association that is to be distinguished from a null association, but when the study results are on hand, there is no longer any need to hypothesize about the magnitude of the association, because you now have an estimate of it. A confidence interval for the estimated association conveys all the relevant information; nothing further is to be gained from a power calculation.

The unfortunate consequence of the focus on statistical significance testing has been to foster a dichotomous view of relationships that are better assessed in quantitative terms. This distinction is more than a nicety. Every day there are important, regrettable and avoidable misinterpretations of data that results from the confusing fog of statistical significance testing. Most of these errors could be avoided if the focus were shifted from statistical testing to estimation.

## CONCLUSION

Why do such important misconceptions about research persist? To a large extent these misconceptions represent substitutes for more thoughtful and difficult tasks. It is simpler to resolve a discrepancy between a trial and a nonexperimental study in favor of the trial, without undertaking the laborious analysis that Hernan et al. did.^10^ It is easy to declare that a result is not statistically significant, falsely implying that there is no indication of an association, rather than to consider quantitatively the range of associations that the data actually support. These misconceptions involve taking the low road, but when that road is crowded with others taking the same path, there may be little reason to question the route. Indeed, these misconceptions are often perpetuated in journals, classrooms and textbooks. I believe that the best prospect for improvement is to raise consciousness about the issues, with reasoned debate. Max Planck once said, “A new scientific truth does not triumph by convincing its opponents and making them see the light, but rather because its opponents eventually die, and a new generation grows up that is familiar with it.”^31^ To the extent that this cynical view is correct, we can expect to see outmoded concepts fade away slowly at best. I hope that calling attention to these misconceptions will spark the needed debates and be a catalyst for change.
